# The Psychology of Testimony and the Interrogation of Children: Contesting the Expertise of Teachers and Female Police Officers, circa 1922–1944

**DOI:** 10.1007/s00048-025-00432-6

**Published:** 2025-10-22

**Authors:** Laurens Schlicht

**Affiliations:** Käte Hamburger Centre for Cultural Practices of Reparation (CURE), Saarland University, Neugrabenweg 4, 66123 Saarbrücken, Germany

**Keywords:** History of psychology, Interrogation practices, History of sexual abuse of children, Women’s movement, Police history

## Abstract

In this case study, I focus on two previously underresearched groups in the history of police interrogation: officers of the Female Criminal Police, established around 1926, and schoolteachers who, beginning in 1924, collaborated with the Leipzig Criminal Office as so-called criminal aides (*Kriminalhelfer*). Both the Female Criminal Police and the criminal aides of the Leipzig Criminal Office claimed a distinct niche within the domain of interrogation practices. They publicly asserted a superior aptitude for questioning children and adolescents, particularly in cases involving the sexual abuse of minors (then classified as Sittlichkeitsdelikte, that is, “moral offenses”) under §176 of the Imperial Penal Code. The article situates these two groups within a broader discourse about interrogation methods that emerged around 1900, a debate increasingly shaped by new psychological approaches—above all, by the emerging field of the psychology of testimony (*Aussagepsychologie*). The question of which epistemic persona could most competently interrogate minors reflected, on one level, professional interests—the pursuit of new occupational opportunities for women and for teachers—and, on another, the contested epistemic authority and social recognition tied to particular forms of subjectivity. In the longer run, female police officers succeeded in establishing their legitimacy because they offered a model that could be integrated into existing police structures: a model of psychologically trained, empathetic officers responsible for cases involving children and young people. Teachers, by contrast, were unable to articulate a comparable epistemic or administrative framework, and their involvement in interrogation practices remained highly localized and short-lived.

“The errors of having pedagogically and psychologically untrained criminal investigators interrogate children are obvious” (Meyer 1925: 61).

At the sixth German Juvenile Court Conference (*Jugendgerichtstag*) in Heidelberg in 1924, the social worker Charlotte Meyer made it unmistakably clear: in her view, the interrogation of children had to be based on a completely new and significantly better kind of knowledge, namely psychology. She was responding to changes in policing and legal practice that had made it possible for new actors to participate in interrogations. This article explores those changes. It uses the example of two groups of actors in the 1920s and 1930s to examine why and how this became possible: a special unit of the German police forces, the Female Criminal Police (FCP), and teachers from the Leipzig Teachers’ Association, who were dubbed “criminal aides” (*Kriminalhelfer*). During the Weimar Republic and under National Socialism, both groups wanted to reshape the interrogation of children, especially in cases of abuse, by drawing on and applying psychological knowledge.

As Antje Schumann has argued, interrogation is located along the “‘interface’ of the state-citizen relationship” (Schumann 2016: 1). It is one of the techniques that states use to interact with their citizens, to learn something about them, and to implement particular modes of state authority. While it is highly controversial as a legal concept, it is clear that interrogation has been, and still is, an authority exercised by the executive and judicial branches of the state—one that is more or less ritualized, formalized, or brutalized. As Eric Engstrom’s contribution to this issue reminds us, and as Elwin Hofman has already shown, the nineteenth century was characterized by a multiplication of knowledge systems and of scientifically trained practitioners who put their knowledge to use in interrogations.^1^

The institutionalization of psychology as a discipline at universities towards the end of the nineteenth century, as well as the introduction of the psychology of testimony (*Aussagepsychologie*) in Germany around 1900, especially by William Stern, enabled the police to exploit and expand the use of new forms of psychological expertise well into the 1920s and beyond (Wolffram 2018). A diverse field of scientists, police officials, teachers, and social reformers became interested in the new psychological methods, not least for reasons of professional advancement. Given that the Imperial Criminal Code (*Reichsstrafgesetzbuch*, RStGB), which came into force in 1871, envisioned interrogation as a matter of public authority, the idea that teachers, psychologists, or social workers should also be able to conduct interrogations was more than just an extension of their professional authority: it fundamentally raised the question of whether and how authorities beyond the executive and judicial branches of government would be allowed to intervene in criminal proceedings. As Wolffram and others have shown, the question of who had enough of the right kind of competence to participate in investigative proceedings or court inquiries was largely about the so-called “boundary work”^2^ or demarcation of different groups of experts from one another. Interrogation reflected only a fraction of the intricate social and epistemic dynamics underpinning this boundary work. Yet interrogation was a very widespread and highly contested terrain, precisely because it had the dual function of being both an executive and judicial technique and, clearly, an epistemic practice. It is precisely for this reason that new experts entered the stage, seeking to take advantage of this jurisdictional ambiguity in order to explore the potential of new scientific alternatives.

In this article I examine the value that officers from the FCP and criminal aides in Leipzig placed on the use of psychological knowledge, how they were involved in assisting the police in their investigations, and how they drew distinctions between themselves and other experts. Comparing teachers and female criminal police officers means comparing two professions that after 1900 made increasing use of forms of academic knowledge. Earlier types of female criminal investigators, such as “police assistants” (*Polizeiassistentinnen*), had already sought to connect psychological and pedagogical knowledge systems, arguing that police work should benefit from this.

Furthermore, both the FCP and criminal aides made use of the new possibilities that arose from the opening up of the juvenile justice system, on the one hand to scientific knowledge in the 1920s, on the other to their respective subject-specific and professional competence. Over the course of this development, especially in the Weimar Republic, the different jurisdictions of the nascent professional institutions had to be carefully balanced, which led the actors to draw on various epistemic and administrative elements, including, as a central element, the practice of interrogation.

Debates in this case revolved largely around the issue of interrogating children in cases of child abuse (§ 176 RStGB) and were very public, very heated, and very politicized (Wolffram 2020). They generated strong opinions because, as the press reported them, two goods worth protecting had to be weighed against each other: the protection of accused but potentially innocent defendants, mostly men, and the protection of children. Psychologists and educators mostly emphasized the protection of the accused, using the principles and techniques of psychology of testimony to discredit the seriousness of children’s allegations (Wolffram 2018; Kerchner 2000; Undeutsch 1954).

This article is comprised of three principal parts. The first part offers an overview of the historical development of the discourse on interrogation (“The Shaping of the Conflict Arena”). In the second part I examine the political underpinnings of interrogation, particularly its role in facilitating the establishment of the FCP (“Gendered Etiquette”). Lastly, in the third part (“Teachers as Psychologists”) I investigate the unique Leipzig institution known as “criminal aides” (*Kriminalhelfer*), a cadre of psychologically trained teachers who collaborated with law enforcement in the investigation of child abuse cases. The concluding section summarizes the preceding discussions and, within the framework of historical epistemology, draws conclusions about how psychology was invoked in policing contexts.

The article is based on sources from the Staatsarchiv Leipzig, the Bundesarchiv Berlin, the Archiv für Diakonie und Entwicklung, Berlin, the Stadtarchiv Regensburg, the Landesarchiv Saarland, as well as on published sources and gray literature.

In order to analyze why specific epistemic-administrative figures were entrusted with the task of interrogating children, one has to examine how interrogation could help craft particular professional profiles and skills. I argue that the ability to convince those in authority of someone’s superior competence in conducting and evaluating interrogations depended on that person’s capacity to publicly justify their epistemic virtues (Daston 2017). In this regard, I draw inspiration from the works of Lorraine Daston and Steven Shapin (Shapin 2008; Daston & Sibum 2003), both of whom have argued that in the history of science we cannot understand how particular individuals could establish themselves as epistemic authorities without an understanding of how these individuals could successfully establish a socially accepted image of their virtues as knowers and producers of knowledge.

Depending on the respective epistemic persona, very different and specific epistemic virtues could be publicly proclaimed. In the realm of interrogation and in the period investigated in this article, I demonstrate the particular significance of two criteria in distinguishing interrogation experts: professional training and subject-specific epistemic virtues, such as the purportedly innate ability of women to establish empathetic contact with children. Both teachers and FCP officers contended that, either by virtue of their education or their innate abilities, they could better engage with children in interrogative settings.

Scholarship on the history of interrogation, on interrogation techniques, and on the legal foundations of interrogation is not extensive. The central legal-historical groundwork for this paper has been done by Schumann (Schumann 2016), Alexandra Ortmann (Ortmann 2014), Alexander Ignor (Ignor 2002), and especially Hofman (Hofman 2022). From the field of criminological and police history, the work of Richard Wetzell, Heather Wolffram, and Annette Mülberger has been key (Wolffram 2020, 2018, 2015, 2009; Wetzell 2017, 2006, 2000; Mülberger 2009a, b, 1994). The contributions of Dirk Götting, Sören Groß, and Ursula Nienhaus have been particularly important for the study of female criminal police (Groß 2021; Götting 2010; Nienhaus 1999a, b). In contrast, the historiography on the professionalization of teachers, their university training, and the role of pedagogy in society is extensive. From this body of literature, the research of Marjorie Lamberti was the most relevant for this article (Lamberti 2002, 2000). There is no specific research on the deployment of teachers in policing, doubtless because this deployment was unique.

Overall, the question posed in this paper is situated at the intersection of the history of science and governmentality studies. However, research on integrating psychological knowledge into areas of governmental practice remains rare and tends to adopt macro perspectives, such as those recently offered by Nikolas Rose (Rose 2007, 1999), while Rose’s earlier works adopted a more fine-grained perspective (Rose 1985). Rose’s argument in his later works is essentially about psychology as a kind of ideological adaptation to new forms of governance and how it contributes to the construction of a new self that is better able to meet the demands of neoliberal economic life. Following turn-of-the-century sociological approaches, but especially Max Weber’s famous “spirit of capitalism,” Rose argues that particular economic systems require particular forms of ideological attitudes on the part of their subjects.^3^

But interrogation, as a technology situated along the interface of scientific aspirations, government practices, and the craftsmanship of policing and judicial process, raises questions that necessitate a return to the genealogical claim of Michel Foucault.^4^ The claim that certain governmental techniques are scientific—such as interrogations or credibility assessments—raises larger and pressing questions about the role of truth, as well as about the historicity in which the legitimacy of scientific systems of knowledge is embedded within those very systems (Kleeberg 2023).

## Shaping the Conflict Arena

Being authorized to conduct interrogations and assess testimony was associated with public recognition. It is therefore hardly surprising that there was a struggle for this very public recognition. As Wolffram and others have shown, in Germany at the beginning of the twentieth century there was an expansion in the field of actors who claimed to be able to exercise authority in policing and law (Wolffram 2020, 2018, 2015, 2009; Ortmann 2014): various actors, from psychologists, psychiatrists, and other medical professionals, to criminologists and teachers, claimed to have something relevant to contribute to the knowledge and practice of interrogation.

Around 1900, psychological insights spurred discussions about the scientific basis of police and judicial interrogation, although for over a century psychology had already been an important resource for legal scholars (Hofman 2022). As many professions, especially those related to social work, increasingly turned to scientific knowledge to undergird their practices (see Sala 2017; Raphael 1996), psychologists began to promote their discipline in the development of interrogation methods. The loudest voice among psychologists was that of William Stern, who in 1906 argued emphatically that “interrogation must be raised from mere routine to a scientific procedure, both for obtaining and evaluating testimony” (Stern 1906: 181). Stern held a preeminent position within the emerging field of psychology of testimony (*Aussagepsychologie*) around the turn of the twentieth century in Germany, expanding upon the earlier research of Alfred Binet in France (compare Clozza 2009: 62–63). Psychologists claimed that they could do two things better than others: assess whether children’s statements in sexual abuse proceedings were truthful, and interrogate them in such a way that the truth of their statements could be ascertained and documented.

The psychology of testimony advocated by Stern and others compelled the teachers and jurists who dealt with children to engage with the claims of psychologists, who were initially few in number. But the psychologists’ networks were expanding. They soon centered around Stern’s intellectual contacts at the Institute for Applied Psychology (Institut für angewandte Psychologie) in Berlin and the Würzburg-based group around Karl Marbe and Maria Zillig (Mülberger 2009a). The psychology of testimony established itself in the 1920s both as an important dimension of debates about child abuse and the training of police officers. More and more decrees and other official regulations stipulated that the psychology of testimony had to be incorporated into the interrogation, whether by using psychological experts or by training judges or public prosecutors. The state of Saxony was one of the pioneers here, as the Ministry of Justice had already stipulated in its ordinance of March 28, 1922, that an expert trained in “sexual psychology” had to be consulted in morality cases whenever they were decided solely on the basis of children’s statements (Döring 1924: 165). Other German states soon followed suit, making the psychology of testimony an essential part of the judicial decision-making process.^5^ Ever since, this kind of psychology has been an inescapable part of debates about interrogation techniques and credibility assessments.

Psychology was taught, among other places, in the Social Women’s Schools (*Soziale Frauenschulen*) founded in the 1910s, thus becoming a field of knowledge for social workers.^6^ These schools were key sites, both for the professionalization of social work and, later on, for demands that women be regularly employed by the police force (Götting 2010). The rather small group of female criminal police officers, supported by the bourgeois women’s movement and some actors in government circles, had been educated at these schools. They had mostly already worked as social workers, and were thus able to put to the test their superior training in scientific subjects compared to most male officers.

Accordingly, the FCP repeatedly emphasized psychological training. For example, in 1931 the FCP officer Grete Henne-Laufer called for a focus on “only those forensic tasks that could be elucidated better by women with a caring disposition and training, underpinned by psychological and pedagogical skills, than by their male colleagues, for whom criminalistic expertise takes precedence.”^7^

In the late 1920s, the work of female police officers became increasingly well-established. Although the total number of female officers remained small compared to male officers, their work was buttressed by more positions, permanent associations, and institutions, including working groups of denominational associations and the women’s movement as organized in the Federation of German Women’s Associations (Bund Deutscher Frauenvereine, BDF; compare Sachße 2003; Schröder 2001). These organizations included the Working Group of German Female Police Officers (Arbeitsgemeinschaft der Polizeibeamtinnen Deutschlands), inspired by the US model.^8^ At a meeting of the group on June 17, 1929, the Dutch women’s rights activist Rosa Manus demanded the “infusion of a feminine, maternal spirit into police work” and the “sublimation of interrogation techniques in accordance with psychological principles.”^9^ Manus echoed the views of most members of the bourgeois women’s movement: like her, figures such as Gertrud Bäumler, Anna Pappritz, and Friederike Wieking, who were instrumental in founding the FCP in Prussia, repeatedly emphasized the importance of femininity, psychology, and interrogation.

The FCP emerged in the 1920s as a product of the efforts of the bourgeois women’s movement (Götting 2010). From the outset, it staunchly supported a subject-centered expertise, asserting women’s unique ability to establish a mental connection with children and adolescents. In the early twentieth century, women initially served as police assistants or police welfare workers for various police forces in the German states (Engstrom 2022). During the Weimar Republic, the first six female criminal police officers began their service in Prussia in 1927, marking the first time that policewomen were officially considered equal to their male counterparts in terms of pay and rank. Women thus successfully contested an area of professional work that had previously been (and would subsequently remain) not only dominated by men, but also very masculine. Establishing their legitimacy in this environment demanded a carefully crafted political and institutional strategy.

The acceptance of this transition within police administrations was facilitated by the strategic efforts of the women’s movement, which worked carefully and deliberately to establish an acceptable figure of the female officer within police forces. Central to this endeavor was the notion that there existed a particular qualification, a “special suitability of women,”^10^ that was shared by most moderate and conservative players in the BDF. This assumption included the belief that, thanks to certain epistemic virtues, women could more effectively elicit testimony from children and adolescents during interrogations and subsequently better assess the veracity of their statements.

Obviously, if one group of actors claimed to be better at a certain task by virtue of a natural talent, another group doing the same task was liable to object. The employees of the Youth Welfare Office (*Jugendamt*), for example, were immediately opposed to this assumption; and for various reasons, another group of professional actors also remained skeptical, namely teachers. Teachers, as Brigitte Kerchner has shown (Kerchner 2000), had from the very beginning adopted a defensive posture and for the most part dismissed accusations of sexual abuse by students. They often based their opposition on the psychological assumption that perfect testimony was rare and that children in particular were highly suggestible and tended to fantasize, especially in the case of sexual topics (Kerchner 1998: 15). However, with the exception of “school reports,” which had to be issued for every case of sexual abuse, teachers were rarely successful in their demands to be included in police investigations.

One of the rare cases in which teachers succeeded in gaining this position occurred in the city of Leipzig. In the 1920s, teachers with psychological training emerged in Leipzig as new experts in interrogations. The director of the Leipzig Criminal Investigation Department, Georg Heiland, issued a series of decrees in the early 1920s that regulated how an interrogation should be conducted in cases of sexually abused children. These decrees generally recommended that interrogations be assigned to a prosecutor rather than the police. Max Döring of the Leipzig Teachers’ Association (Leipziger Lehrerverein) was of the opinion that the involvement of teachers would be appropriate if a public prosecutor was not immediately available and the involvement of the police was unavoidable (Döring 1924: 182).

It was the specific history of their profession that led teachers to claim to be competent interrogators or interrogation assistants. At the beginning of the twentieth century, for example, the Berlin teachers’ association advocated that children should first be interrogated by teachers before coming into contact with the judicial system or the police forces (Mohr 1909: 63). Powers akin to executive authority had already been exercised by teachers, as they had long been responsible for maintaining school discipline. In situations not dissimilar to court proceedings, part of this duty involved disciplining students, administering penalties, and ascertaining the truth. The tradition of “school courts” (*Schulgerichte*) and the common practice of questioning students likely convinced teachers of their inherent ability to assess adolescents with whom they interacted on a daily basis.^11^

It was the development of criminal procedural law in Germany (and other countries) that made interrogation, free reporting, situational tactics, and new interrogation specialists possible. As Ignor notes, there had long been precise rules about how to conduct an interrogation—rules involving written procedures designed to establish the legal “truth.” As he further argues, it was primarily social changes that forced courts to adapt criminal procedure (Ignor 2002: 57).

One of the central legal norms in Germany was § 136 of the Imperial Code of Criminal Procedure (*Reichsstrafprozeßordnung*, 1877). The paragraph stipulated (and still stipulates) that the purpose of the interrogation was to give the accused the opportunity to present facts in their favor, to apprise them of the charges against them prior to the interrogation, and to instruct them that they were obliged neither to testify nor to testify truthfully. These and other rules helped ensure that many older truth-seeking techniques, such as trickery, coercion, or technical devices (like the lie detector), became more difficult or impossible to use. What was true for the interrogation of the defendant extended a fortiori to the interrogation of witnesses and experts. Lawyers, police officers, medical experts, and jurors all had to rely on the evaluation of testimony, especially in trials where there was little or no other evidence. It is therefore hardly surprising that a psychology of testimony attracted considerable interest among jurists and the police.

Reformers hoped to open up interrogations to new experts outside the traditional realms of administrative and legal practice. This initiative inherently gave rise to conflicts, prompting Stern, Marbe, and other proponents of the psychology of testimony to place significant emphasis on establishing cordial relations with interested legal practitioners.^12^ Examined from a sociological perspective, the subsequent coalition between legal professionals and psychologists can be interpreted as a reciprocal resource relationship (Ash 2002): the emerging academic discipline of psychology benefited by diversifying its scope, while the jurists gained ground by scientifically substantiating their traditional professional authority.

## Gendered Etiquette, Tact, and Psychological Prowess: Female Criminal Police Interrogations

Women have been assisting police work since the early twentieth century, serving as police assistants (*Polizeiassistentinnen*), police welfare workers (*Polizeipflegerinnen*), as well as jail and prison nurses (*Polizei‑, Gefängnisschwester*) (Götting 2010). However, prior to the establishment of auxiliary police (*Gefährdetenpolizei*) and the Female Criminal Police (*Weibliche Kriminalpolizei*), they were never formally equal to their male counterparts within the police force. In 1926, in Prussia and a few other German states, this situation changed when the first women began being trained for the police force. This represented the culmination of a long period of political mobilization by the bourgeois women’s movement involving various actors.^13^

The legal foundations for the FCP were laid in the 1920s in various ordinances and decrees (see Groß 2021). Although these measures cannot be analyzed in detail here, it must be emphasized that the right to conduct interrogations was an essential part of the FCP’s demands from the very beginning. In this respect, the establishment of the FCP in Prussia and several other German *Länder *helped codify a practice that had previously been and still was common in various cities. In 1928, for example, the police social worker in the small village of Schneidemühl reported that she too conducted investigations and interrogations into moral offenses.^14^

The FCP evolved out of police assistants, who were often engaged in charity work. Although advocates of the FCP insisted that they were not charity workers, their work often differed *de facto* little from that of assistants, who, as shown, were already conducting interrogations. Emphasizing that female officers now explicitly conduct interrogations as police officers must be read as a shift in professional politics rather than any profound change in the daily practice of police work. The claim that practices and responsibilities had fundamentally changed served to make it abundantly clear to the outside world that, although the new institution of the FCP might outwardly resemble the police assistant, it was institutionally, praxeologically, and professionally completely new. In fact, many members of the FCP had previously worked as police assistants and usually continued to perform the same tasks.^15^ Proponents of the Female Police saw it as a model for an intuitive, situational, and minimally invasive police force. In their vision of a “modern” police force, interrogation methods had moved beyond the idea of violent coercion. In the words of the Cologne-based FCP officer Elfriede Dinger about interrogation at a conference at the Police Institute (Polizei-Institut Charlottenburg) in 1926:

“Inspired by daily practice, the open-minded and responsible police officer will inevitably have to develop concepts that serve the dual purposes of objective truth-finding and youth protection. She must engage with the physical and mental makeup of the young individuals and continuously orient herself within the realms of psychology, pedagogy, criminal law, and criminology. Only then will she approach her mission of objective truth-seeking.”^16^

Leading voices promoting the FCP also insisted that the intangible notion of “tact” could be acquired through psychological training. This kind of “tact” was intended to underscore that a very specific persona was needed to initiate contact with children in interrogation settings: it was related to both gender and class differences. In a groundbreaking essay in 1930, Josephine Erkens stated that any “psychological and social” analysis of crime required the necessary “psychological sensitivity and tact, and the conscious pedagogical and psychological methods to overcome all inhibiting and obscuring influences, especially during interrogation” (Erkens 1930: 75). The “tact” of experts conducting interrogations has long been part of discussions about interrogation tactics in medical and legal contexts, as Engstrom’s article (this issue) shows. Subsequently, female police officers continued to use the term “tact” to refer to the elusive ability that FCP officers had to have. In her Berlin “Guide” to the work of FCP officers (an internal report from 1937 on the occasion of the tenth anniversary of the female police department in Berlin), Heide Gobbin remarked that the interrogation of young people required “a special degree of comprehension, skill, and tact.”^17^ She emphasized that “the officer’s most important concern” was “to establish contact with the child.”^18^

Gaining the authority to interrogate was an integral part of the initiatives in the 1920s and a key demand of Josephine Erkens (1889–1974; see Nienhaus 1999a, b). Erkens was head of the FCP in Frankfurt am Main, a Prussian city at the time, which in addition to Berlin was one of only two training centers for the FCP in Germany. She was forced to resign after 1933 due to her sympathies for the Social Democratic Party during the Weimar Republic (Groß 2021: 245). In Frankfurt in 1926, after the training of the first FCP officers had already begun, she drafted “General Principles for the Allocation of Duties to a Female Police Force and its Placement in the Police Department.”^19^ In that text she explained that “the women’s police will investigate crimes that are suitable for elucidation by female, socially and psychologically trained officers. The aim is to ensure objective truth-finding as well as a comprehensive assessment of the individual’s personality in legal proceedings involving juveniles and women.”^20^

In her text, Erkens referred to the newly enacted Juvenile Courts Act (*Jugendgerichtsgesetz*, 1923) and the National Child Welfare Act (*Reichsgesetz für Jugendwohlfahrt*, 1922), as well as various ordinances and decrees, which required that the “personality” of juveniles be evaluated in proceedings against or involving them. Assessing “personality” became an especially important task, since it involved assessing “domestic circumstances,” a task which was actually the prerogative of the Youth Welfare Office (*Jugendamt*), which had just been created in 1922 and which objected to the police encroaching on its newly acquired responsibilities.^21^ So, while female police officers had to position themselves against the Youth Welfare Office when investigating domestic circumstances, they claimed the authority of the public prosecutor or the male criminal police when interrogating. Female police officers, according to Erkens, should

“[develop] a complete picture of [the juveniles’] mental structure for the purpose of testing the *credibility* of their testimonies. However, the nature and thoroughness of the statement will depend primarily on the empathy and tact of the interrogator. Children and adolescents will only open up fully to individuals in whom they have complete trust, allowing them to provide insights that are conducive to clarifying the facts. In the case of juvenile and female defendants, a more comprehensive assessment of their personality from psychological and social perspectives can be obtained, serving as a foundation for sentencing and the personalized imposition of penalties.”^22^

Interrogations required, as Erkens said, “special sexual-pedagogical sensitivity” to avoid harming children and adolescents. She continued: “Furthermore, … interrogations of women and girls in cases involving moral offenses [*Sittlichkeitsdelikte*] require a distinct approach that understands the nature of women.”^23^

Erkens’s argument integrated the two central assumptions that had also been foregrounded in political discourse about female police officers: the gendered epistemic competence and “nature of women” on the one hand, and their pedagogical and psychological training on the other.

The emphasis on these two characteristics was by no means accidental and gave rise to conflicts that needed to be managed for political and professional reasons. Compared with their male counterparts, female police officers benefited from longer and better training as social workers at a women’s social welfare school, complemented by coursework in criminal investigation.^24^ But to judge by contemporary press reports, FCP officers were generally successful in defusing tension with their male colleagues. Most press reports reinforced the belief that women could interrogate children and adolescents better thanks to their “femininity,” “empathetic understanding,” or their ability to “place themselves in the minds of others.” The following quote from a press article of 1934 underscores the central importance of interrogation:

“The tactical criminal interrogation is indeed one of the most challenging tasks. It is not uncommon for juveniles to be brought in as witnesses in cases involving sexual offenses. One involuntarily thinks, “How can these statements help me,” when faced with such a small child! However, even these children can often provide important information if questioned correctly. Their mental development must always be taken into account, and it must be ensured that no one else has previously asked them suggestive questions.”^25^

The quote, extracted from a 1934 report on the Women’s Police in Gdansk, then “Danzig,” illustrates how the discourse on interrogation remained unchanged after 1933. Additional newspaper reports up until the 1940s and into the postwar period confirm that this constancy endured. Only rarely does one find statements from female police officers that deviate from the preferred narrative of a balance of power and directly question the competence of their male colleagues. For example, the officer Kall from the Frankfurt FCP remarked at a conference of female police at the Police Institute in Berlin in 1931, that when men interrogated men, they often overlooked “sociopsychological aspects.”^26^

Typically, female officers’ activism also involved preserving the precarious balance that ensured the survival of the numerically small German FCP (59 female police officers in Prussia in 1927; 162 in 1932; 207 in 1945 for the whole Reich). FCP officers were very deliberate in their actions and paid careful attention to how they were perceived by the general public.

During the Great Depression, the three denominational professional organizations for female police established a working group that reported on how leaders felt they should proceed in dire economic times. The working group was convinced that, for the foreseeable future, the number of female police officers would increase only slowly, if at all. In the opinion of the working group, it was up to the interest groups to emphasize the need for and benefits of the FCP.^27^ Proponents of female police officers again reiterated their two long-standing arguments about the “special aptitude” (*besondere Eignung*) of women and their psychological skills during interrogations. In the opinion of the working group, however, it was no longer enough “to repeatedly assert the ‘special suitability of women’ and their ‘maternal,’ ‘intuitive,’ and other ‘qualities,’” for now “successes” were needed: “For instance, we should have highlighted our achievements as acknowledged experts in child interrogations and juvenile offenses as a whole.”^28^ The report went on to diagnose that interrogations in the FCP remained too unsystematic and were too often based on the local habits of individual officers. Thus, despite professional training by the police and familiarity with the psychology of testimony, interrogation practices had not yet reached the level of professionalism needed for the public to recognize that female police officers were the better interrogators.

**Fig. 1:**
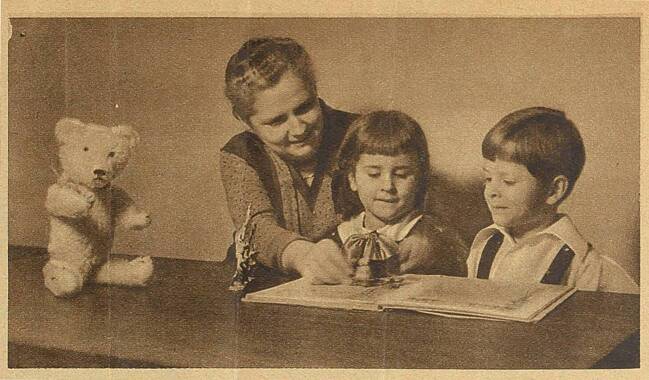
A Female Criminal Police Officer Prepares Children for Interrogation

The tactic of women’s associations and the BDF was thus to create a credible and politically effective *persona* that enabled both activists and police organizations to view the participation of women in police work as complementary rather than competitive. Accordingly, the proponents of the FCP had to do a lot of rhetorical work to contain the competition that actually existed. Focusing on interrogation was an effective choice, since it could draw on publicly accepted stereotypes that had also been mobilized in the FCP’s public relations efforts. A newspaper article from 1934, for example, portrayed a sympathetic image of the female interrogator. The article was richly and carefully illustrated and showed the interrogator concerned about the child’s welfare. One of the photographs showed how the female officer prepared children for the interrogation. In accordance with police regulations, the female investigator was dressed in civilian clothes. She engaged with the children, playing with them at first, while a teddy bear gazed quizzically at the observer (Fig. 1).

In addition to the positive tactic of emphasizing the benefits of female officers, proponents of the female police pursued the negative strategy of enumerating the areas in which women would not participate in police work. For example, an article in *Die Frau* in 1929 stated that women would “under no circumstances” be entrusted with detective work and convictions, since the public rightly felt an “aversion” to such activities when carried out by women.^29^ Using this approach, female officers aimed to ease the apprehensions of their male colleagues, largely by refraining from direct physical involvement in what were considered “unladylike” tasks.

The evolution of juvenile criminal law in the 1920s and under National Socialism were favorable for the FCP in terms of decrees and ordinances. The idea of fighting juvenile crime prophylactically rather than repressively had long been a key demand of the nineteenth-century criminal law reform movement (*Strafrechtsreformbewegung*) (Wetzell 1996). The basic idea of this kind of reform program was to “render harmless” those who were judged to be “incorrigible criminals.” The policing of crime therefore needed to be based on accurate knowledge about the population. Given its socio-scientific and psychological outlook, the FCP fit perfectly into this concept of policing. Not least thanks to this tradition, it was expanded from 1935 onward, first in Prussia and then from 1937 across the Reich; proponents worked actively to achieve the goals of National Socialist crime policy (Groß 2021; Nienhaus 1999a; Neugebauer 1997). Among other things, the FCP’s deployment in youth concentration camps, where one of their central tasks was the interrogation of juveniles, is currently being investigated.

The prospects for FCP officers seeking to assert their influence over criminal interrogations were good because they were doubly talented as “women” and “psychologists.” Crossing the lines demarcating personality and ability, they valued both. In particular, they emphasized the value of scientifically informed, subject-centered competence in determining the truth of children’s testimony. They were successful because they carefully avoided offending male officials and were able to deal successfully with other competing institutions, especially the Youth Welfare Office.

### Leipzig Teachers as Psychologists and “Criminal Aides” (*Kriminalhelfer*)

**Fig. 2:**
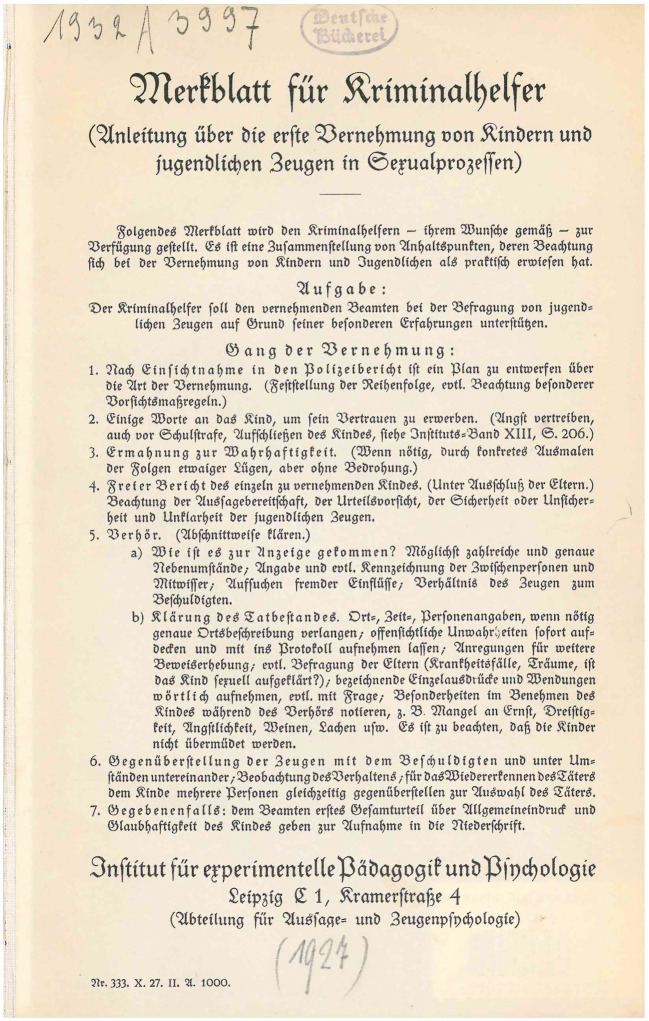
Merkblatt für Kriminalhelfer, 1927 [Translation provided in Appendix 1]

The leaflet in Fig. 2 responded to the need of teachers, trained by the Leipzig Teachers’ Association (LLV) to work as so-called “criminal aides,” for guidance in the interrogation of children and adolescents. It had been developed by the Association’s Division for the Psychology of Testimony and Witnesses. The Association was a powerful organization with a mostly progressive orientation during the Weimar period, when it established its own institute for experimental psychology and pedagogy. The use of teachers as “criminal aides” was unique and did not survive the National Socialist era. As will become clear below, the use of criminal aides was made possible because Leipzig had established a remarkably cooperative relationship between certain parts of the police force and local psychological and pedagogical institutions. Psychologically trained teachers succeeded in projecting a professional persona that combined academic training and practical knowledge.

In what follows, I draw on both published and archival materials to trace selected dimensions of the practical involvement of criminal aides in the context of police interrogation. I do not aim to reconstruct a coherent or stable institutional practice, but rather to foreground one possible instantiation of a contested and ultimately unsuccessful attempt to establish an alternative expert system within the practice of interrogation. It must be assumed that other variants of interrogation work coexisted in Leipzig, and there is little reason to posit a uniform mode of teacher involvement across cases. Whether the practices of criminal aides ever amounted to a systematized form of expertise remains, from today’s perspective, uncertain. This uncertainty is itself a product of the archival documentation in the State Archives of Saxony (Sächsisches Staatsarchiv): there exists no record of which cases were removed from the archive, on what grounds, how extensive the original corpus may have been, or how frequently criminal aides were consulted. In light of these archival limitations, I looked for cases involving charges under § 176 of the Imperial Penal Code, paying particular attention to the involvement of officers from the Female Criminal Police or criminal aides. This was enabled—unlike in other archival settings—by the highly searchable holdings of the State Archives in Leipzig (Staatsarchiv Leipzig). Still, neither completeness nor representativeness can be claimed.

Unlike the FCP, teachers as interrogators never became a viable model of success, but instead remained a local exception made possible in the early 1920s thanks to various ministerial decrees in Saxony. For example, a decree issued by the Saxon Minister of the Interior on May 6, 1922, stipulated that children were to be interrogated only once and by a public prosecutor “specially trained in the psychology of child testimony.”^30^ A subsequent regulation, the Saxon Supplemental and Executive Ordinance (*Sächsische Ergänzungs- und Ausführungsverordnung*) of July 9, 1923, stipulated that psychological experts should be consulted if a defendant denied the accusation of sexual abuse made by a juvenile witness. It explicitly stated that: “The Institute for Experimental Pedagogy and Psychology, Leipzig, Kramerstraße 4 may also be consulted in the selection of experts” (quoted in Plaut 1931: 287). In 1924, the head of Leipzig’s criminal investigation department, Gerhard Heiland, began using criminal aides, deepening the influence of psychological knowledge on local police work.^31^ In an article about this unusual arrangement, Heiland remarked:

“On June 7, 1924, an agreement was reached between the Institute for Psychology of the Leipzig Teachers’ Association and Leipzig’s Criminal Investigation Department. According to this agreement, whenever criminal investigators question juvenile witnesses, teachers with special pedagogical-psychological training would be called upon to serve as honorary criminal aides. They also have the right to question the child. Both the questions and answers are recorded in the court transcript. After completing the interrogation, the aides are usually able to provide an initial overall assessment of the child’s general impression and credibility, which is of the utmost value for further investigation” (Heiland 1926: 40).

The fact that such an arrangement was possible had to do with Leipzig’s special status. Not only had Wilhelm Wundt established the first psychological university laboratory there in 1879, but Max Brahn had held the first chair of experimental pedagogy. The first institute for experimental psychology created by teachers was affiliated with the LLV and had been founded in 1906 with the help of Wilhelm Wundt (Taubert-Striese 1996). This institute became a model for a number of other similar institutions established in Berlin, Breslau, Chemnitz, Dresden, Hamburg, and Munich (Lamberti 2000: 40). Just as police assistants had the authority to conduct interrogations on their own, criminal aides could also interrogate individuals as long as they maintained good relations with the police.

Although designed to provide teachers with some guidance without over-determining their involvement, it is unclear how closely the text of the above leaflet (Fig. 2) ultimately reflected the actual practices of the criminal aides. Nevertheless, it is clear that the collaboration of the Criminal Investigation Bureau with the LLV fostered a mutually supportive relationship. The leaflet outlined an interrogation situation in which, ideally, a police officer and an expert representing the LLV were present, and in which the expert’s role was essentially to listen and assess the testimony.

In fact, criminal aides were also much more directly involved in the interrogation situation than the 1927 leaflet suggests. Richard Wetzel, in particular, can serve as an example. Wetzel was senior teacher at an auxiliary school (*Hilfsschuloberlehrer*), an LLV member, and a zealous advocate of teachers as psychological experts. A member of the Division for the Psychology of Testimony and Witnesses at the Institute for Experimental Psychology and Pedagogy, he frequently served as a criminal aide, published extensively on the subject of children’s interrogations, and was well connected with researchers investigating the psychology of testimony.^32^ Wetzel exemplified a number of teachers who conducted research and published on psychological topics. These teachers exploited the opening of universities to elementary teachers and the teaching of psychology in teacher training colleges and *Soziale Frauenschulen* to enhance their opportunities for socio-epistemic advancement.^33^ Wetzel continued to work for the Leipzig Criminal Investigation Department after the LLV was disbanded by the National Socialists and reorganized as a National Socialist institution.^34^

A case of child abuse from 1939 can help illustrate some of the jurisdictional disputes that arose in relation to interrogations and juvenile law. Although retired at the time, Wetzel had continued to work for the police as a criminal aide. In May 1939, together with the chief prosecutor, he participated in an interrogation at the inn “Zum alten Fritz” in Engelsdorf near Leipzig.^35^ Wetzel was present during the prosecutor’s interrogation and also asked questions of the witnesses and the injured woman. In his June report, however, Wetzel emphasized the importance of making a conceptual distinction between the “interrogation” and his “questioning,” presumably so as not to arrogate an authority that was not his.^36^ As in all cases involving children and juveniles, the student’s school also submitted an expert report. The structure of this and other similar reports had been standardized in the 1920s by the LLV’s Institute for Experimental Psychology and Pedagogy; the categories and terminology used were drawn from developmental psychology and the psychology of testimony.^37^ In Wetzel’s case, we find criminal aides accompanying the authorities and at times intervening during interrogations, but also maintaining a low profile in the reporting of these events.

But other cases show that LLV criminal aides could use their psychological skills much more assertively, defending their own opinions against other authorities in criminal proceedings. For example, in 1940, senior teacher Albert Stein gave a psychological assessment of a juvenile witness in an abuse case.^38^ Stein not only observed the witness during questioning, but also applied psychological tests for intelligence, fantasy, suggestibility, eideticism, and memory that had been further developed by the LLV’s Institute for Experimental Psychology and Pedagogy. Although the intelligence test in this case yielded a below-average score, Stein reported that the witness gave “an overall impression that is not unfavorable.”^39^ While the witness may have had limited “conceptual-logical abilities,” he also displayed a sort of “shrewdness” and could sufficiently observe, combine, and remember things, showing no “eccentricities” in his imagination.^40^ Nor was the witness overly suggestible, an important observation because, ever since Alfred Binet’s *La suggestibilité *(Binet 1900), suggestibility had been at the center of debate in testimonial psychology and was considered to be one of the major threats to accurate testimony. In his expert opinion, Stein concluded that the witness was credible, thus reaching a conclusion that contradicted the FCP’s assessment in the case. The police officer who interrogated the witness in November 1939, approximately a month before Stein’s evaluation, had a “very unfavorable impression” of the witness. Specifically, the witness appeared “stubborn” and “had a foolish, vacant look.”^41^ Officer Magdalene Eales^42^ concluded that the juvenile witness was primarily reproducing what his mother had previously instilled in him. In other words, he was under the dangerous dominion of suggestion.

These are just two of many cases. But nevertheless, some general conclusions can be drawn from them. First, criminal aides were indeed able to use psychological testing methods.^43^ Although the methods employed, such as intelligence testing, were not usually specified, it was at least possible in Leipzig’s criminal justice system to integrate psychological methods into the practice of interrogation, in collaboration with the police. However, secondly, the practice of assessing witnesses was not so standardized as to dictate what conclusions the aides should draw from their tests. Ad hoc assumptions and individual reflections enabled the criminal aides to still advocate for the credibility of witnesses even in the presence of relatively negative test results. In the case cited above, Stein referred to the witness’s “cunning,” unquestionably a non-psychological term, which he introduced to convey a particular impression of the witness. Further research is needed to determine to what extent the assessment practices of the criminal assistants differed structurally in their results (credible or not) from the assessments of other authorities.

The use of criminal aides in Leipzig is an example of an unsuccessful initiative. But it is worth asking why this model, in contrast to the FCP, failed to establish itself. Lacking sufficient documentary evidence, perhaps it is permissible to infer some conclusions from the better-documented professional strategies of the FCP. The (relative) success of the FCP was due to the fact that it was able to collaborate with other actors in a way that was acceptable to all participants. It conceded greater authority to male colleagues in other areas and thus was seen as less of a threat. Although its relations with the Youth Welfare Office were contentious, the FCP was able to manage those relations successfully because of the professional persona of the officer, who combined tact with psychological training. As a result, the FCP created a professional niche for itself that was specific enough to be sustainable without fundamentally challenging the claims of other actors.

The teachers were unable to do this. Their professional persona left the other actors, especially the police, basically no room to differentiate their own interrogative skills from those of teachers. Teachers claimed to be better trained, to have closer contact with and better knowledge of the children, and they could even point to their own psychological training. Experience conducting interrogations, as the article by Wieser (this special issue) shows, was often a marker used to document proficiency, and police officers in particular very often cited their experience. Teachers, however, did the same. Although further research is needed, some of the reasons for the failure of the criminal aides can be inferred from the success of the FCP. Furthermore, from a sociology of knowledge perspective, it is already well documented in similar contexts that successful professional boundary work was a key factor in the assertion of certain epistemic virtues.

## Conclusion

With the introduction of the Juvenile Court Act (1923) and the National Child Welfare Act (1922), the legal foundations were laid for the development of an expert-based criminal justice policy for juveniles and children. The epistemological prerequisites for implementing this science-based policy had been created since 1900 with the growth of child and adolescent psychology and the psychology of testimony, which helped integrate scientific knowledge into criminal law and social welfare programs. Whether this actually led to an improvement in the ability to judge whether statements made during interrogations are credible cannot be determined retrospectively. A 2014 study of US police officers found that they were more likely than not to be convinced that they could detect lies. However, they actually did no better than the general population in a randomized control group study, correctly detecting lies only about 50 percent of the time, that is, not detecting them at all (Hartwig et al. 2014). The evidence-based and experimental procedures established as the standard for testimonial psychology reports since a 1999 Federal Court ruling may well offer enhanced empirical validity (Steller & Volbert 1999; Fiedler & Schmid 1999). However, it is crucial to acknowledge that the assessment of their efficacy is contingent upon the epistemic framework of the psychology of testimony. An in-depth analysis of this matter, unfortunately, lies beyond the scope of the present discussion.

What this article has shown, however, is that since the 1920s there has been a proliferation of epistemic personae who claim to be able to judge the testimony of children and adolescents. Two of these new groups of experts have been examined here: teachers serving as criminal aides and officers of the Female Criminal Police; a third, the Youth Welfare Office, has been mentioned. Exactly what this proliferation means for the history of science, and how the subject of interrogation finds its place in this history, deserves further study.

The right to interrogate had to be claimed and supported by credible arguments. The opening of the legal system to new fields of knowledge and to new kinds of experts also elicited dispute and forced the new epistemic and administrative personae to demonstrate the effectiveness of their science-based competence as well as their natural or acquired inalienable abilities. Officers from the FCP were ultimately successful because they were convincing in both respects. As empathetic, psychologically trained interrogators, they were able to carve out a space for women in the police force. This history of emancipation, which led to the permanent deployment of women as officers in the criminal police forces in the years after 1926, found them mostly supporting the National Socialist regime, to which they put up very little resistance. Teachers were not able to establish themselves as criminal aides on a permanent basis. One explanation for this can be found in the instable boundary work with the other groups involved in criminal investigations: if teachers were indeed the better interrogators, then other authorities, who were less well trained and had less contact with children, were potentially always in danger of having their competence questioned in a fundamental way.

The right to conduct and evaluate interrogations is no less a question of power: Who has the right to determine the truth of statements and to question the most vulnerable members of society, namely children? Who should receive more protection in abuse trials, children or the accused? As this article has shown, these questions of power were woven into a complex web of competing interests, with different groups assessing the truth of statements and the truthfulness of those making them in specific ways. Behind the dense layer of representations that we find in protocols, reports, scientific and popular publications, and in the web of different and sometimes polemically expressed opinions, it is no longer possible to reconstruct what originally happened during interrogations, let alone which side was right. However, historical epistemology can reconstruct the way in which different groups—in this case FCP officers and teachers as criminal aides—entered the field as new experts, what role psychological expertise played, and how it functioned in each case. These functions are historically diverse; this article has focused on the professional-political dimension of psychological training.

## Abbreviations

ADE: Archiv für Diakonie und Entwicklung: Archive for Social Welfare Work and Development
BDF: Bund deutscher Frauenvereine: Federation of German Women’s Associations
LLV: Leipziger Lehrerverein: Leipzig Teachers’ Association
RStGB: *Reichsstrafgesetzbuch*: Imperial Penal Code

## Footnotes

1 The reference to different forms of knowledge by different interrogators is currently being investigated and still offers many unexplored avenues for further research. Elwin Hofman shows that already in the early nineteenth century, actors from the fields of jurisprudence and law enforcement referred to psychology as a “useful science.” He argues that the history of psychology should therefore also deal with these early forms of psychological knowledge in practical contexts (Hofman 2022: 330). The history and function of the interrogation is presented in Schumann (2016) and Ignor (2002).

2 Gieryn originally defined the term to mean the demarcation between science and non-science: “Boundary-work is analyzed as a rhetorical style common in ‘public science’ …, in which scientists describe science for the public and its political authorities, sometimes hoping to enlarge the material and symbolic resources of scientists or to defend professional autonomy” (Gieryn 1983: 782). However, Gieryn left open the possibility that the term could also be used to analyze processes of demarcation within the sciences. Wolffram then suggested that the term be used generally to refer to the boundaries between different professional fields (Wolffram 2018). This is exactly how the term is used below.

3 Rose reads Weber affirmatively and reproduces him as follows: “The Protestant revolution begins a new era in the culture of the self and the systems for self-direction, in which the union of conscience, casuistry, and the cure of souls is rejected; in its place, each individual comes to bear the obligation of doing the will of God without the benefit of learned confessors, directors, and advisors” (Rose 1999: 224).

4 In *Governing the Present*, Rose and Miller themselves argue that the “genealogy of identity” must draw on Foucault’s genealogical approaches (Rose & Miller 2008: 24). Foucault developed his ideas on genealogy as a method of historical critique in various texts. In “Qu’est-ce que la critique” (1978) he summarized some of these ideas, discussing genealogy as a method of historical critique and how it relates to efforts to identify causal relations in history. According to Foucault, instead of producing definitive causalities, genealogical methods enable one to imagine causality as a “network” that has made possible certain “effects.” Foucault wants to understand both the systems (the “networks”) that have made certain singularities possible, and the relationship of these singularities to the historical succession of those systems (Foucault 1990 [1978]). This approach essentially aims to understand the interconnectedness of relatively permanent macrostructures with relatively changeable micro-practices.

5 Mostly in the early 1920s, other German states also made consultation with either psychological experts or at least psychologically trained lawyers mandatory in cases of sexual abuse. Cf.: Erste preußische Rundverfügung 11.11.1903; Zweite preußische allgemeine Verfügung über die Vernehmung jugendlicher Personen im Ermittlungsverfahren, 18.12.1920; Braunschweigische Verfügung betr. Vernehmung von Schulkindern als Zeugen in einem Strafverfahren wegen Sittlichkeitsverbrechens oder Vergehens, 13.5.1921; Erste sächsische Verordnung, die Vernehmung jugendlicher Zeugen in Sexualprozessen betreffend, 28.3.1922; Württembergischer Erlaß an die Staatsanwaltschaften, 22.12.1922; Bayerische Bekanntmachung über die Behandlung der Strafsachen wegen Verbrechen und Vergehen wider die Sittlichkeit gegenüber Jugendlichen, 9.1.1923; Anhaltische Verfügung (see the 2. preußische Verfügung), 19.4.1923; Zweite Sächsische Verordnung zur Ergänzung und Ausführung der Verordnung vom 28.3.1922, 9.7.1923; Rundschreiben von Mecklenburg-Schwerin über die Vernehmung weiblicher Minderjähriger im Strafverfahren wegen Verbrechens oder Vergehens gegen die Sittlichkeit, 10.7.1923; Allgemeine Thüringische Verfügung über die Vernehmung jugendlicher Zeugen in Strafverfahren wegen Sittlichkeitsverbrechen und -vergehen, 18.1.1924 (Döring 1924: 168).

6 The importance of women’s social schools for the history of women’s social work was examined by Schröder (2001). She also underscored the relevance of psychology in women’s social schools (ibid., 252–53).

7 ADE, ADW, CA, Gf/St, 318, Bericht über die Tagung der weiblichen Polizei am 9. und 10. Mai 1931 in Berlin, Polizei-Institut Charlottenburg.

8 In 1915, the International Policewomen’s Association was founded in the USA, which was the model for other associations of its kind.

9 “Frauen-Berufe—Weibliche Polizei”, *Korrespondenz Frauenpresse*, April 17, 1930.

10 Lothar Barck summarized the tasks of the female police in a widely read comprehensive survey as follows: “the essence and goal of the female police are the same everywhere: the pursuit of a social effect of the police based on the special psychological aptitude of women” (Barck 1928: 39).

11 Mohr mentioned this institution in her study about the way children were being treated by courts. She pointed out that judges who dealt with juvenile cases (*Jugendrichter*) would mostly treat children more kindly than the old “school courts” (*Schulgerichte*) (Mohr 1909: 12).

12 See the volume edited by Matthias Schmoeckel (Schmoeckel, ed. 2009).

13 For an authoritative account of this mobilization, see especially Götting (2010).

14 ADE, ADW, CA Gf/St, 317, Bericht über die Tätigkeit der Polizeifürsorgerin in Schneidemühl.

15 Some of the FCP’s most important players had previously been police assistants, including Friederike Wieking, the FCP’s most powerful officer during the Nazi era.

16 ADE, ADW, CA, Gf/St, 318, Report by Dr. Schr on the meeting of the three associations held June 16–17, 1926, at the Prussian Police Institute.

17 FFBIZ, papers of Rotschuh, B Rep. 500, acc. 400, 10.3, “Wegweiser durch die Arbeit der Dienststelle KJ. M III 2,” 1937.

18 Ibid.

19 ADE, ADW, CA Gf/St, 316, Erkens: Allgemeine Grundsätze für die Zuteilung der Aufgabengebiete an eine weibliche Polizei und ihre Einordnung in die Polizeibehörde; n. d., probably 1926 or 1927.

20 Ibid.

21 Dickinson describes how the establishment of the Youth Welfare Office sparked a debate on the question of whether it should be staffed by specialists in the field of social pedagogy or rather by traditional administrative staff (Dickinson 1996: 172). Overall, the question of how the authority of the Youth Welfare Office should be exercised and structured was the subject of lively debate.

22 ADE, ADW, CA Gf/St, 316, Erkens: Allgemeine Grundsätze für die Zuteilung der Aufgabengebiete an eine weibliche Polizei und ihre Einordnung in die Polizeibehörde; n. d., probably 1926 or 1927.

23 Ibid.

24 The principles governing the training of female criminal police officers are described in Groß (2021).

25 “Die Frau in der Polizei—Aus der Tätigkeit eines Danziger weiblichen Kriminalbeamten,” *Danziger Vorposten*; n. d., presumably 1934.

26 ADE, ADW, CA, Gf/St, 318, Bericht über die Tagung der weiblichen Polizei am 9. und 10. Mai 1931 in Berlin, Polizei-Institut Charlottenburg.

27 ADE, ADW, CA Gf/St, 145—Weibliche Polizei, AG der Fachgruppen Weibliche Polizei an die Mitglieder der AG, Breslau, June 10, 1931.

28 Ibid.

29 *Die Frau*, no. 5, March 13, 1929.

30 Quoted in Plaut (1931: 286).

31 Heiland was then dismissed in 1933 for political reasons and was appointed as a judge to the Federal Constitutional Court after the war in 1951. He endorsed William Stern’s psychology of testimony and documented in various texts his conviction that the integration of psychology into police work was important (Heiland 1929, 1926).

32 On his views about the interrogation of children, see Wetzel (1930). An informal discussion about the function of psychological experts is documented for the year 1928, in which Wetzel took part alongside Otto Lipmann, Paul Palut, Hermuth Bogen, Curt Bondy, William Stern, Max Döring, and Walter Hoffmann (Anonymous 1928: 266).

33 On the history of the “Soziale Frauenschulen,” see Sachße (2003); Zeller (1994).

34 The Institute for Experimental Psychology and Pedagogy was transformed into a “Fachschaft” of the National Socialist Teacher’s Association (*Fachschaft des Nationalsozialistischen Lehrerbundes*).

35 SächsStA, StA‑L, 20114, Landgericht Leipzig, Nr. 6904, Oberstaatsanwalt, Leipzig, May 17, 1939.

36 Ibid., Wetzel’s expert report on the statements of juvenile witnesses, Leipzig, June 11,1939.

37 Döring (1924: 187–88) specified what information a school report had to contain. Döring’s detailed regulations also dealt with teachers’ psychological assessments, such as the “moral and psychological credibility” of a child or their “susceptibility to influence.”

38 SächsStA‑L, 20114, Landgericht Leipzig, 6885, expert report of the head teacher Albert Stein, January 4, 1940.

39 Ibid.

40 Ibid.

41 Ibid., Interrogation by the FCP, Nov. 27, 1939.

42 The following archival source provides some information about this officer: SächsStA, Sta‑L, 20031, PP‑M 1745.

43 An expert report by a senior teacher from 1940, for example, states: “The present expert opinion is based on the study of the files and on a psychological examination of [the witness] with subsequent questioning on the matter,” SächsStA, StA‑L, 20114, 7015, expert report of the senior teacher Krimmling, November 30, 1940. The report makes it clear that Krimmling used various methods to test aptitude (presumably intelligence), memory and retention, and imagination.
